# Left-ventricular hypertrophy in 18-month-old donor rat hearts was not associated with graft dysfunction in the early phase of reperfusion after cardiac transplantation–gene expression profiling

**DOI:** 10.1007/s11357-021-00348-8

**Published:** 2021-04-19

**Authors:** Sevil Korkmaz-Icöz, Deniz Akca, Shiliang Li, Sivakkanan Loganathan, Paige Brlecic, Mihály Ruppert, Alex Ali Sayour, Andreas Simm, Maik Brune, Tamás Radovits, Matthias Karck, Gábor Szabó

**Affiliations:** 1 Laboratory of Cardiac Surgery, Department of Cardiac Surgery, University Hospital Heidelberg, 69120 Heidelberg, Germany; 2 Department of Cardiac Surgery, University Hospital Halle (Saale), 06120 Halle, Germany; 3 Heart and Vascular Center, Semmelweis University, 1122 Budapest, Hungary; 4 Department of Medicine I and Clinical Chemistry, Heidelberg University Hospital, 69120 Heidelberg, Germany

**Keywords:** Left-ventricular hypertrophy;, Heart transplantation;, Spontaneously hypertensive stroke-prone rats;, Ageing;, Gene expression

## Abstract

The use of hearts with left-ventricular (LV) hypertrophy (LVH) could offer an opportunity to extend the donor pool for cardiac transplantation. We assessed the effects of LVH in 18-month-old spontaneously hypertensive stroke-prone (SHRSP) donor rats and following transplantation. In donors, cardiac function and structural alterations were assessed. Then, the hearts were transplanted into young normotensive-rats. We evaluated LV graft function 1 h after transplantation. The myocardial expression of 92 genes involved in apoptosis, inflammation, and oxidative-stress was profiled using PCR-array. Compared to controls, SHRSP-rats developed LVH, had increased LV systolic performance (slope of the end-diastolic pressure-volume (PV) relationship: 1.6±0.2 vs 0.8±0.1mmHg/μl, *p*<0.05) accompanied by diastolic dysfunction [prolonged time constant of LV pressure decay (Tau: 15.8±0.6 vs 12.3±0.5ms) and augmented diastolic stiffness (LV end-diastolic PV relationship: 0.103±0.012 vs 0.045±0.006mmHg/ml), *p*<0.05]. They presented ECG changes, myocardial fibrosis, and increased nitrotyrosine immunoreactivity and plasma troponin-T and creatine kinase-CM levels. After transplantation, even though the graft contractility was better in SHRSP rats compared to controls, the adverse impact of ischemia/reperfusion-injury on contractility was not altered (E_es_ ratio after versus before transplantation: 32% vs 29%, *p*>0.05). Whereas nitrotyrosine immunoreactivity was higher, myeloperoxidase-positive cell infiltration was decreased in the SHRSP+transplanted compared to control+transplanted. Among the tested genes, LVH was associated with altered expression of 38 genes in donors, while transplantation of these hearts resulted in the change of four genes. Alterations in 18-month-old donor hearts, as a consequence of hypertension and LVH, were not associated with graft dysfunction in the early phase of reperfusion after transplantation.

**Supplementary Information:**

The online version contains supplementary material available at 10.1007/s11357-021-00348-8.

## Introduction

Heart transplantation is the current curative treatment option for end-stage heart failure. However, the imbalance between waiting lists and surgery rates continuously increases due to higher demand with no increase in the supply of suitable organs. Therefore, adequate and optimal utilization of the donor pool is essential. Efforts have been made to expand donor acceptance criteria by using so-called “appropriate marginal” donors, who would be declined under conventional transplant guidelines. The concept of marginal donors consists of donors who are older, have hepatitis C virus-positivity, a history of alcoholism, diabetes mellitus, an ejection fraction <45%, or a donor/recipient weight ratio <0.7 [5]. Nevertheless, the most common criteria causing a donor heart to be rejected include cold ischemia >4h, donor age >55 years, left ventricular (LV) hypertrophy (LVH) >1.3 cm, and LV ejection fraction ≤50%.

One potential approach is the inclusion of hypertrophied donor hearts harvested in the compensatory phase of hypertrophy [8]. Currently, hearts with LVH are not included in donor selection guidelines. LVH is a maladaptive response of the heart to chronic pressure overload. The myocardial morphological and electrophysiological changes, occurring to overcome a pressure overload, can be divided into three stages: developing hypertrophy, compensatory hypertrophy, and overt heart failure [6]. As a brief summary, we can declare that short-term adaptive cardiac hypertrophy becomes maladaptive in the long-term and may lead to further cardiac damage. Aortic valve stenosis, systemic hypertension, and artherosclerosis are clinical conditions associated with pressure-overload [21]. Systemic hypertension is defined as a repeatedly elevated blood pressure with a systolic pressure above 140 mmHg and/or a diastolic pressure higher than 90 mmHg [30]. It should be noted that more than one third of the population is affected in developed societies; therefore, patients with hypertensive cardiomyopathy could demonstrate a notable donor pool for heart transplantation [7]. Furthermore, the prevalence of LVH and abnormal LV geometry increases with age [14, 15]. However, only sporadic data exists regarding the use of donor hearts with LVH in cardiac transplantation [8, 19] and experimental investigations evaluating the impact of LVH on both post-transplant graft function and molecular screening are still limited to date. Goland et al. have demonstrated that selected hearts from donors with mild and moderate LVH can be safely used for heart transplantation and may increase the number of hearts available for transplantation [8]. Wever et al. have showed that the use of allografts with LVH in association with other high-risk characteristics may result in increased mortality [31].

The stroke-prone spontaneously hypertensive (SHRSP) rat is a sub-type of spontaneously hypertensive (shr) that shows severe hypertension with high incidence of cerebral stroke attacks [23]. These rats are hypertensive at 5 weeks of age, and the hearts of 6- to 12-month-old spontaneously hypertensive rats have been reported to have well-compensated LVH [9, 29]. To investigate the effects of both ageing and LVH, hearts from 18-month-old rats were used in the present study.

Taking this into consideration, we assessed the effects of LVH in 18-month-old SHRSP donor rats and following transplantation. In order to further identify molecular alterations occurring in the LVH donor heart before and after transplantation, gene expression changes associated with inflammation, apoptosis, and oxidative stress were investigated.

## Materials and methods

### Animals

Male SHRSP rats (Charles River, Sulzfeld, Germany) and age- and sex-matched normotensive Wistar rats were housed in a room at 22±2°C under 12-h light/dark cycles, and were fed a standard rodent regime with water ad libitum. The final experiments on the rats were performed at an age of 18 months. The animals received humane care in compliance with the “Principles of Laboratory Animal Care” formulated by the National Society for Medical Research, and with the “Guide for the Care and Use of Laboratory Animals,” prepared by the Institute of Laboratory Animal Resources and published by the National Institutes of Health (NIH Publication No. 86-23, revised 1996). This study was approved by the appropriate institutional review committees (G128/15).

### Normotensive and spontaneously hypertensive stroke-prone donors

#### Experimental groups

The rats were divided into two groups: (a) control donor rats and (b) SHRSP donor rats (*n*=6–8 rats/group).

#### Echocardiography

One day prior to the final experiment, the rats were slightly anesthetized with 1.5–2.0% isoflurane by mask, the left side of the chest was shaved to obtain a clear image, and the animals were situated in the supine position on a warming pad. Transthoracic echocardiography was performed by using an HDI 5000 CV echocardiography machine (ATL Ultrasound, Philips, Bothell, WA, USA) equipped with a 10-MHz linear probe. Two-dimensional parasternal short-axis images as well M-mode recordings at the mid-papillary muscle level were assessed. The following parameters were measured: left-ventricular (LV) internal end-diastolic dimensions during diastole. LV mass was calculated to estimate the myocardial weight using the Devereux formula: LV mass (g) = {[(LVEDD + AWT_d_ + PWT_d_)^3^ – LVEDD^3^]×1.04}×0.8 + 0.14 [4]. To exclude an influence of body weight differences, these parameters were normalized to body weight. In addition, LV volumes were estimated according to the Prolate method: LVEDV= [(3.14/6) × LVEDD^2^] × L [25]. The quantitative analysis of the LV systolic function consists of fractional shortening (FS), calculated as [(LVEDD-LVESD)/LVEDD] × 100, LV ejection fraction, and cardiac output/index.

#### Electrocardiography

Before the hemodynamic measurements, the rats were anesthetized with sodium pentobarbital (60 mg/kg, i.p.) and kept in a supine position on heating pads, maintaining their core temperature (measured via a rectal probe) at 37°C. As previously reported [11, 13], standard 12-lead electrocardiograms were recorded using subcutaneously placed needle electrodes.

#### In vivo left-ventricular cardiac function: pressure volume analysis

After the ECG recordings, as previously reported [11, 13], the rats were tracheotomised, intubated, and artificially ventilated with ambient air. A polyethylene catheter was inserted into the left external jugular vein for fluid administration. A 2F-microtip pressure-volume catheter was inserted into the right carotid artery and advanced into the ascending aorta. After a 5-min stabilization period, the arterial blood pressure was recorded, and the catheter was advanced into the left-ventricle under pressure control. With the use of a special pressure-volume-analysis program (PVAN, Millar Instruments, Houston, TX, USA), heart rate, systolic (SBP) and diastolic blood (DBP) pressures, mean arterial pressure (MAP), maximal slope of systolic pressure increment (dP/dt_max_) and diastolic pressure decrement (dP/dt_min_), and time constant of the LV pressure decay (Tau-g; according to the Glantz method and Tau-w by Weiss method [24]) were calculated. LV pressure-volume relations were assessed by transiently compressing the inferior vena cava. The slope of the LV end-systolic pressure-volume relationship (ESPVR) was calculated according to the linear (E_es_) and the parabolic curviliniear model (E_max_) [10]. The slope of the LV end-diastolic pressure-volume relationship (EDPVR) was calculated as a reliable index of LV stiffness.

#### Biochemical analysis

After hemodynamic measurements were completed, blood samples from the abdominal aorta were collected. After centrifugation (4,500g, 15 min, 4°C), plasma samples were obtained. The levels of high-sensitive cardiac troponin-T and creatine kinase-MB were determined in the central laboratory of the Heidelberg University clinic with ECLIA on Cobas E411 (Roche Diagnostics).

#### Histopathology

After the blood sample collection, all rats were sacrificed by bleeding and the hearts were explanted. Pieces of the LV myocardial tissue were fixed in buffered paraformaldehyde solution (4%) and embedded in paraffin. Then, 5-μm thick sections were stained with hematoxylin and eosin. Cardiomyocyte cross-sectional areas were calculated on a microscope using the Cell^A software (Olympus Soft Imaging Solutions GmbH, Germany). An acid fuchsin orange G (AFOG)-stain was used to determine the extent of myocardial fibrosis. Four sections per heart were inspected under light microscopy and rated according to the following scoring system: grade 0 indicates normal tissue showing no fibrotic region; grade 1 indicates mild fibrosis; grade 2 indicates moderate fibrosis, and grade 3 indicates severe fibrosis. The histological evaluation was conducted by an analyst unaware of the experimental groups.

### Rat model of heterotopic heart transplantation

#### Experimental groups

The rats were divided into two groups: (a) control+transplanted group received hearts from normotensive donors and (b) SHRSP+transplanted group received hearts with LVH. The hearts was transplanted into young Sprague-Dawley rats (*n*=7–13 rats per group).

#### Surgical technique of heart transplantation

Transplantations were performed from Wistar to Sprague-Dawley and SHRSP to Sprague-Dawley rat strains. The experimental model was described elsewhere [13, 16]. Briefly, cardiac arrest was induced via Custodiol solution (Dr. Franz Köhler, Chemie GmbH, Bensheim, Germany), then the heart was explanted and immediately placed in Custodiol solution (4°C). Recipient Sprague-Dawley rats were anaesthetized and then heparinized. The aorta and the pulmonary artery of the donor heart were anastomosed end to side to the abdominal aorta and the vena cava of the recipient rat, respectively. To minimize variability between surgical experiments, the duration between explantation and reperfusion was standardized to 1 h. After completion of the anastomoses, the heart was reperfused with blood in situ for 1 h.

#### Functional measurement in the graft

As previously reported [12], 1 h after transplantation a 3F latex balloon catheter (Edwards Lifesciences Corporation, Irvine, CA, USA) was introduced into the left-ventricle via the apex and connected to a precision-calibrated syringe for administration and withdrawal of fluid. Additionally, a Millar micromanometer (Millar Instruments, Houston, TX, USA) was inserted in the left-ventricle to determine LV systolic pressure, developed pressure, dP/dt_max_ and dP/dt_min_, LVEDP, and Tau at different LV volumes. LV volumes were calculated as the volume of saline injected into the balloon plus the volume of the empty balloon (0.02 ml). Data for a complete pressure-volume curve were obtained through incremental increases in LV volume by 0.03 ml until a volume of 0.17 ml was reached.

### Gene expression analysis

Using RT^2^ Profiler^TM^ PCR Array, the expression of 92 genes was profiled before and following heart transplantation. The official names of these genes are presented in (online Table 1). These genes were selected as they have been reported to be key genes involved in inflammation, apoptosis, and oxidative stress. Total RNA was extracted from LV myocardial samples with a miRNeasy Mini Kit (Qiagen, Hilden, Germany) and was reverse-transcribed into cDNA using the RT^2^ First Strand Kit, mixed with RT^2^ qPCR Master Mix, containing SYBR Green, according to manufacturer's instructions (Qiagen, Hilden, Germany). In this Custom Array, the following non-regulated genes (genes-of interest) were used for normalization in the fold change expression data calculations: beta-2 microglobulin (B2m) and hypoxanthine-guanine phosphoribosyl transferase-1 (Hprt1). Genes with fold regulation greater than 2 or less than -2 at *p*<0.05 were considered as significantly altered.

### Immunohistochemistry

LV myocardial tissue samples were fixed in buffered paraformaldehyde solution (4%) and embedded in paraffin or stored in -80°C until they could be cut into frozen sections. Then, blocks were cut into 5-μm-thick paraffin or frozen sections. The immunoreactivity to myeloperoxidase (MPO) (1:100; Abcam, Cambridge, UK) and nitrotyrosine (1:100; Abcam, Cambridge, UK) was investigated. Infiltrating neutrophils (MPO-labeled) were counted, and nitrotyrosine expression was semi-quantitatively assessed based on staining intensity and the distribution of the labelled target protein. Furthermore, we performed terminal deoxynucleotidyl transferase-mediated dUTP nick end-labeling (TUNEL) staining to detect DNA-strand breaks as described previously [13–15]. The number of TUNEL-positive cells was expressed as the ratio of DAPI-TUNEL double-labeled nuclei to the total number of nuclei stained with 4’, 6-diamidino-2-phenylindole (DAPI). Each specimen recieved an average score of four adjacent fields in a blinded fashion.

### Statistical analysis

All data is expressed as mean ± standard error of the mean (SEM). Statistical analyses of data were performed using GraphPad Prism 7.02 software (GraphPad Sofware, Inc., CA, USA). Before statistical tests were applied, the Shapiro-Wilk and D'Agostino-Pearson normality tests were used to assess normal distribution. For data with normal distribution, two-sample Student *t*-test was used to analyze the differences between the two groups. If the normality test failed, a nonparametric Mann-Whitney *U*-test was applied. In the case of PCR-Array gene expression, the *p*-values were calculated based on a Student’s *t*-test of the replicate 2^(-Delta Ct) values for each gene in the experimental groups. A value of *p*<0.05 was considered indicative of statistical significance.

## Results

### Detailed characterization of experimental model of LVH in 18-month-old donors

#### Body weight, heart and lung weights, cardiomyocyte diameter, and myocardial collagen accumulation

**Fig. 1:**
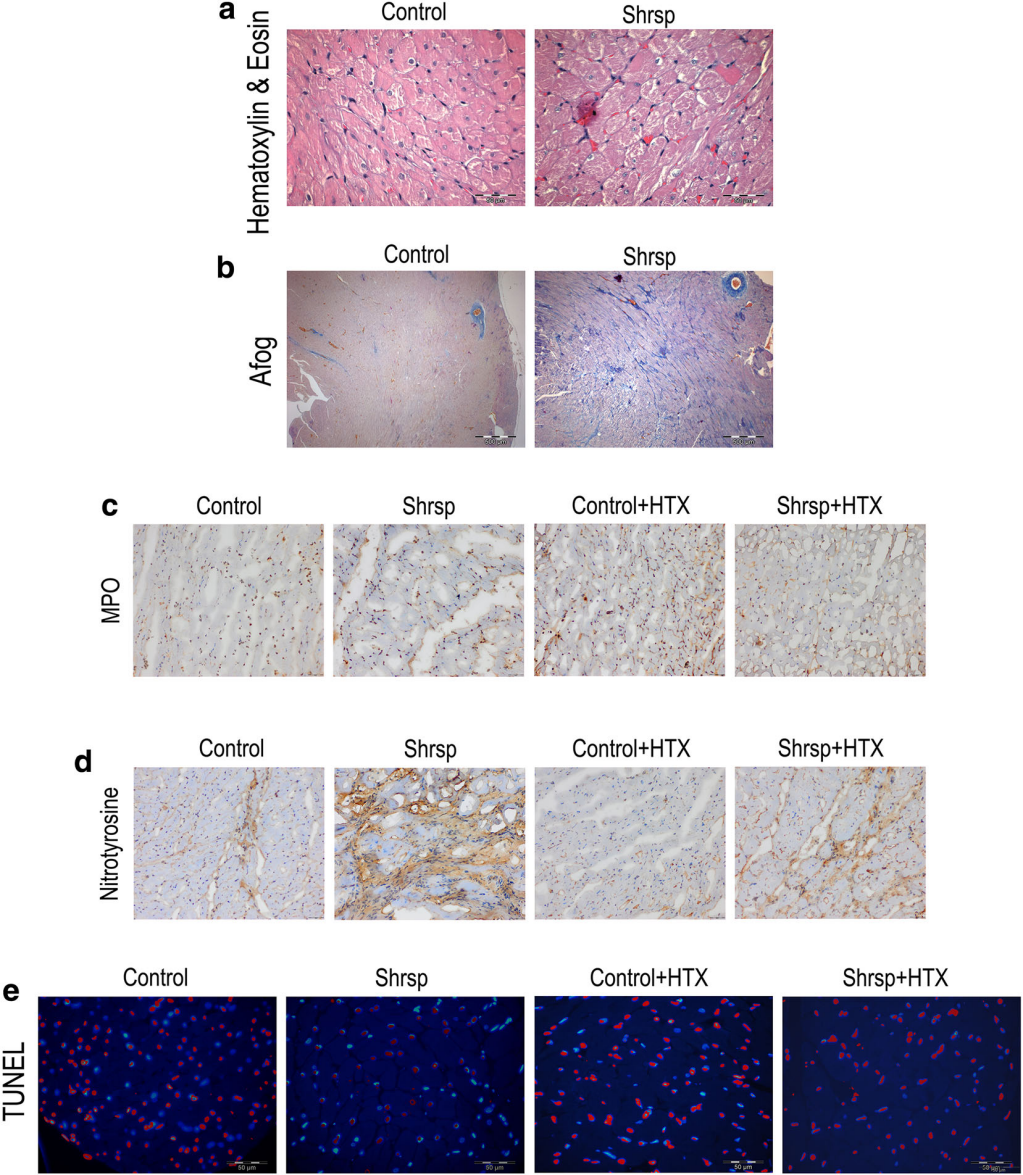
Representative photomicrographs of myocardium in donors and after transplantation. (A) hematoxylin and eosin (×400, scale 50 μm), (B) acid fuchsin orange G (Afog) (×100, sclae 500 μm), (C) myeloperoxidase (MPO) (×200, scale 50 μm), (D) nitrotyrosine (×200, scale 50 μm), and (E) terminal deoxynucleotidyl transferase-mediated dUTP nick end-labeling (TUNEL) (×400, scale: 50 μm) in control and SHRSP rats. SHRSP indicates stroke-prone spontaneously hypertensive rats and HTX, heart transplantation

**Fig. 2:**
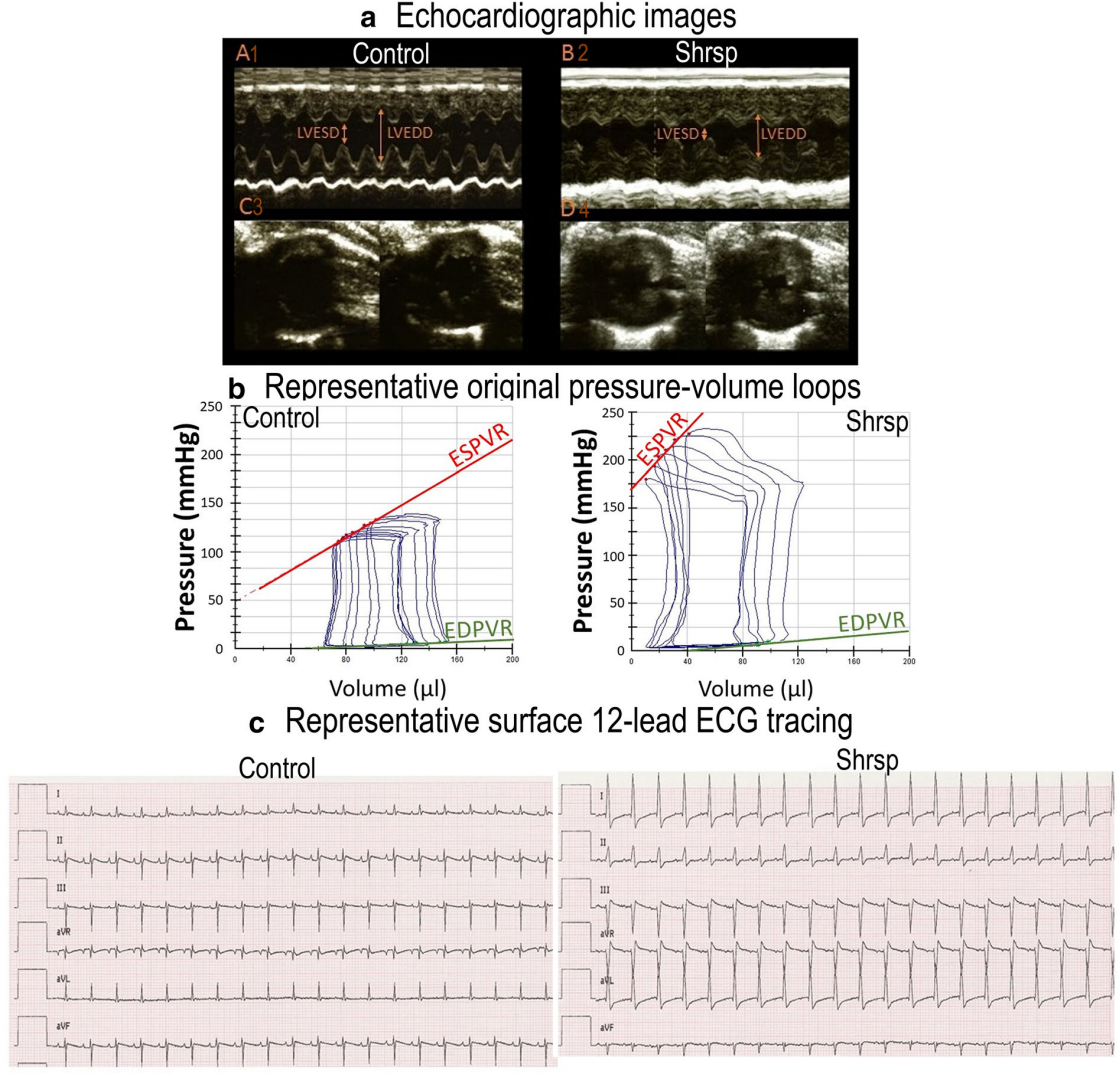
Representative echocardiogram, pressure-volume loops, and surface electrocardiogram (ECG) in donors. (A) Representative two-dimensional echocardiographic images (A1–B1) M-mode recordings and (C1–D1) short axis in control and SHRSP rats. (B) Representative pressure-volume loops obtained with a micromanometer conductance catheter system at different preload. End-systolic pressure-volume relationship (ESPVR) and end-diastolic pressure-volume relationship (EDPVR) in one representative animal in control and SHRSP groups. (C) Representative surface ECG tracings in control and SHRSP rats. SHRSP indicates stroke-prone spontaneously hypertensive rats; LVESD, left-ventricular end-systolic dimension; LVEDD, left-ventricular end-diastolic dimension

Compared to controls, SHRSP rats develop LVH, which was confirmed by significantly increased heart weight-to-body weight ratio, increased cardiomyocyte diameter normalized to body weight, and increased LV mass index. Additionally, excessive myocardial collagen accumulation in SHRSP rats indicated myocardial fibrosis. Furthermore, a decreased body weight, heart weight and lung weight, and an increase in lung weight-to-body weight was observed in the SHRSP rats compared to controls (Table 1, Figs. 1A–B and 2).

**Table 1:** Body weight, heart weight, heart weight/body weight ratio, lung weight, lung weight/body weight ratio, cardiomyocyte diameter normalized to body weight, and myocardial collagen accumulation

	Control	SHRSP	*p* value
Body weight [g]	661 ± 22	322 ± 4*	<0.0001
Heart weight [g]	2.03 ± 0.10	1.56 ± 0.02*	0.0004
Heart weight / body weight x1000	3.09 ± 0.14	4.85 ± 0.10*	<0.0001
Lung weight [g]	1.98 ± 0.07	1.43 ± 0.06*	<0.0001
Lung weight / body weight x1000	3.00 ± 0.11	4.45 ± 0.17*	<0.0001
Cardiomyocyte diameter / body weight [μm*1000/g]	25.4 ± 0.8	67.7 ± 2.4*	<0.0001
Collagen accumulation (AFOG staining, score: 0-4)	0.78 ± 0.1	1.64 ± 0.2*	0.0112

*SHRSP* spontaneously hypertensive stroke-prone rats, *AFOG* acid fuchsin orange G**.** *P<0.05 versus Control. *n*= 6–7 rats/group

#### Neutrophil infiltration, nitro-oxidative stress, and DNA strand breaks

Immunoreactivity for nitrotyrosine was significantly higher in the SHRSP group compared to controls (score: 4.50±0.50 versus 3.25±0.56, *p*<0.05). However, the number of MPO-positive cell infiltration (60±4 versus 64±4 positive cells, *p*>0.05) and the number of TUNEL-positive nuclei (70±5 versus 65±4%, *p*>0.05) were not was significantly altered between SHRSP and control groups (Fig. 1C–E).

#### Echocardiographic parameters

**Fig. 3:**
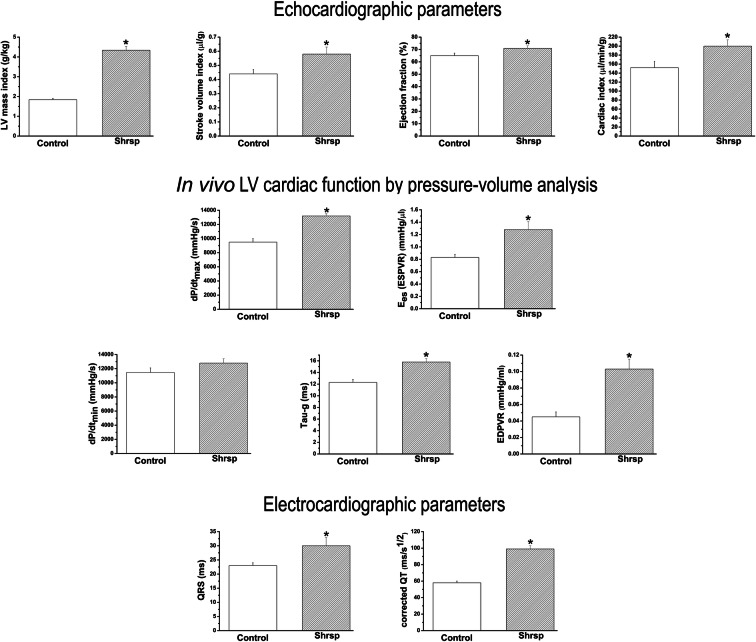
Echocardiographic parameters, in vivo left-ventricular (LV) cardiac function by pressure-volume analysis, and electrocardiographic parameters in donors. SHRSP indicates spontaneously hypertensive stroke-prone rats; Tau-g, time constant of LV pressure decay (according to the Glantz method); ESPVR, end-systolic pressure-volume relationship; EDPVR, end-diastolic pressure-volume relationship. *P<0.05 versus Control. *n*= 19-22 rats/group

Compared to controls, echocardiographic parameters displayed significantly increased stroke volume index, ejection fraction, and cardiac index in SHRSP rats (Table 2, Figs. 2A and 3).

**Table 2:** Echocardiographic parameters, in vivo left-ventricular cardiac function by pressure-volume analysis, and electrocardiographic parameters in donors

	Control	SHRSP	*p*-value
Echocardiographic parameters			
Heart rate [beats/min]	342 ± 16	347 ± 51	0.81
LV end-diastolic diameter [mm]	7.17 ± 0.15	5.51 ± 0.15*	<0.0001
Fractional shortening [%]	38.0 ± 1.7	41.5 ± 1.5	NS
LV end-diastolic volume [μl]	449 ± 25	264 ± 15.5*	<0.0001
LV end-systolic volume [μl]	157 ± 13	75 ± 3.9*	<0.0001
Stroke volume [μl]	292 ± 17	189 ± 14.4*	<0.0005
Cardiac output [μl/min]	100888 ± 8656	64962 ± 3945*	<0.05
In vivo LV cardiac function by pressure-volume analysis			
Systolic blood pressure [mmHg]	137 ± 5	221 ± 7*	<0.0001
Diastolic blood pressure [mmHg]	111 ± 4	166 ± 5*	<0.0001
Mean arterial pressure [mmHg]	120 ± 4	184 ± 5*	<0.0001
Heart rate [beats/min]	369 ± 9	321 ± 7^*^	0.0001
Tau-w [ms]	10.7 ± 0.6	11.8 ± 0.4	NS
E_max_ (ESPVR) [mmHg/μl]	2.75 ± 0.18	5.92 ± 0.62*	<0.001
Electrocardiographic parameters			
Heart rate [beats/min]	342 ± 16	347 ± 10	NS
RR [ms]	178 ± 9	174 ± 5	NS
PR [ms]	43 ± 2	48 ± 2	NS
QT [ms]	58 ± 3	93 ± 3*	<0.0001

*LV* left-ventricular, *Tau-w* time constant of LV pressure decay (according to the Weiss method), *ESPVR* end-systolic pressure-volume relationship, *SHRSP* spontaneously hypertensive stroke-prone rats. *P<0.05 versus control. *n*= 19–22 rats/group

#### Hemodynamic parameters

Compared to controls, SHRSP rats had increased LV systolic performance (including indices of load-dependent (maximum rate of rise of left-ventricular pressure dP/dt_max_ and cardiac index, ejection fraction) and enhanced load-independent (the slopes E_es_ and E_max_ of the end-systolic PV relationship) parameters in the presence of diastolic dysfunction [(prolonged time constant of LV pressure decay Tau) and increased diastolic stiffness (slope of the end-diastolic PV relationship)] (Table 2, Figs. 2B and 3).

#### Electrocardiogram patterns

On ECG recording, compared to controls, SHRSP rats displayed a significant increase in QRS complex duration and prolongation of corrected QT (Table 2, Figs. 2C and 3).

#### Biochemical parameters

Plasma levels of cardiac Troponin-T (401±107 vs 89±29 pg/ml, *p*=0.0173), and creatine kinase-MB (370±60 vs 166±16 U/I, *p*=0.006) were significantly increased in SHRSP rats compared to the control group.

### Effect of LVH on graft function after transplantation

**Fig. 4:**
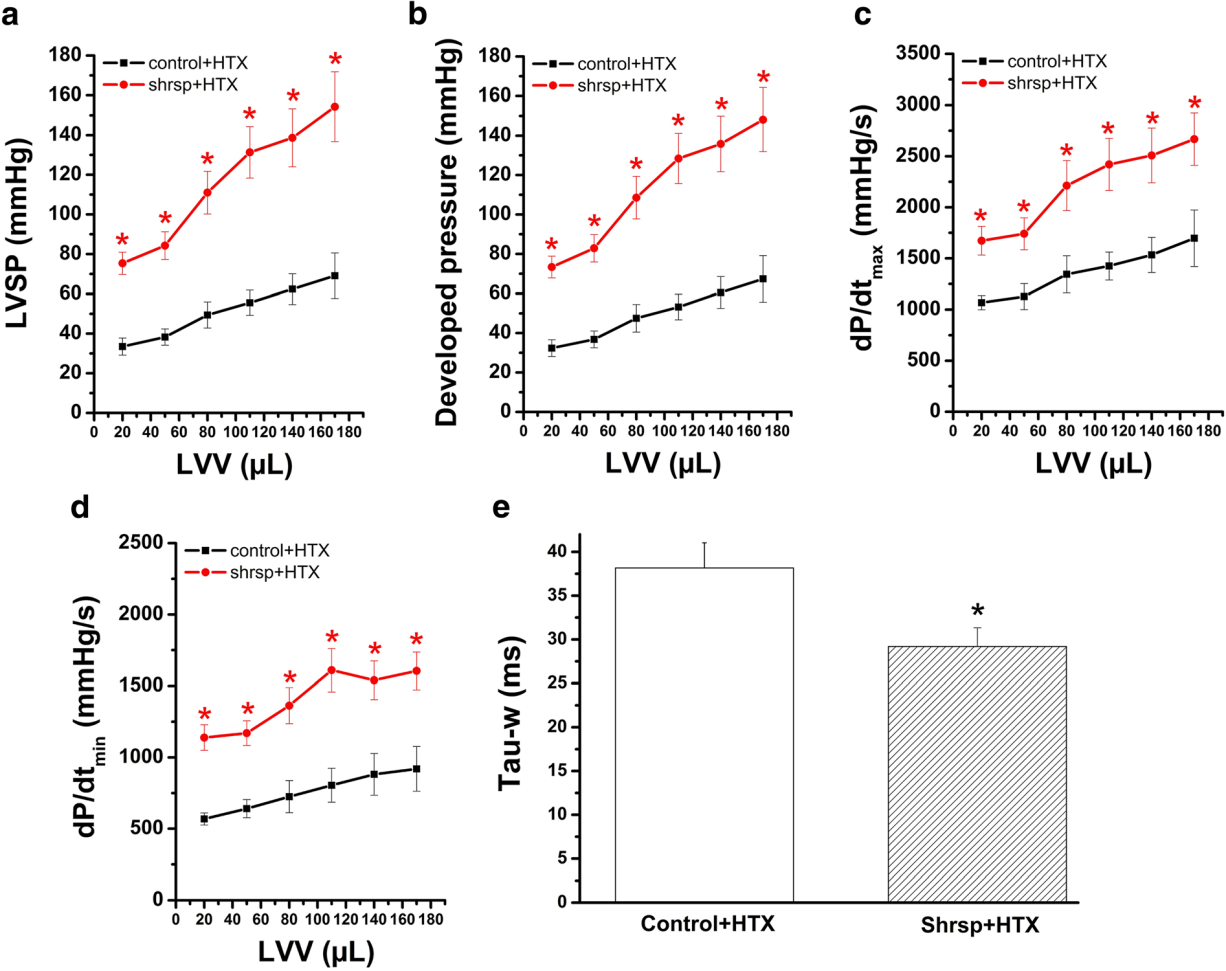
In vivo left-ventricular (LV) graft function after heart transplantation (HTX). SHRSP indicates spontaneously hypertensive stroke-prone rats; LVSP, LV systolic pressure; dP/dt_max_, maximum rate of rise of left-ventricular pressure; dP/dt_min_, maximum rate of fall of left-ventricular pressure; LVEDP, LV end-diastolic pressure; Tau, time constant of LV pressure decay (at an intraventricular volume of 50 μl); and LVV, LV volume. *P<0.05 versus Control. *n*= 7–13 rats/group

After heart transplantation, significantly increased LV systolic pressure, developed pressure, and dP/dt_max_ were observed in the SHRSP hearts when compared with the control group, indicating an increased systolic function (Fig. 4A–C). Furthermore, dP/dt_min_ was significantly higher and Tau-w was significantly decreased in the SHRSP group compared to controls (Fig. 4D–E). Even though graft contractility was better in SHRSP rats compared to controls, the adverse impact of ischemia/reperfusion injury on contractility was not altered (E_es_ ratio after to before transplantation: 32% versus 29%, *p*>0.05).

### Effect of LVH on neutrophil infiltration, nitrotyrosine, and DNA-strand breaks after transplantation

Immunohistochemical analysis shows that LVH significantly decreased MPO-positive cell infiltration (49±5 versus 64±5 positive cells, *p*<0.05) and increased nitrotyrosine immunoreactivity (score: 3.46±0.49 versus 2.13±0.26, *p*<0.05) in the SHRSP+transplanted group compared to the control+transplanted rats (Fig. 1). After transplantation, DNA-fragmentation, as reflected by an increased number of TUNEL-positive nuclei was observed in the control+transplanted versus control (75±4 versus 65±4%, *p*<0.05) and in the SHRSP+transplanted versus SHRSP (83±2 versus 70±5%, p<0.05) (Fig. 1).

### Changes in cardiac gene expression caused by LVH in donors and their implication in heart transplantation

#### SHRSP donor heart versus normotensive control donor heart

To determine the effects of LVH-induced cardiac changes, 92 genes in the donor heart were surveyed. Among them, the expression of 38 genes were significantly altered in SHRSP hearts compared to controls (2 genes were upregulated: *Gpx1*, *Ccl20* and 36 genes down regulated: *Akt1, Aox1, Apafl1, Bcl2l1, Birc3, Casp1, Casp2, Casp4, Casp8, Cat, Ccl24, Ccl3, Ccl4, Ccl5, Ccr2, Ccr3, Cd401g, Epx, Fas, Fasl, Il13, Il15, Il16, Il3, Il4, Il7, Il9, Mpo, Ncf1, Nos2, Noxo1, Tgfb1, Tnf, Tnfrsf10b, Tnfsf10, Tpo,* Table 3, first column).

**Table 3:** Fold-regulation and associated *p*-value of all tested genes. For all significantly altered genes, fold-regulation values are presented in red. See online Table 1 for genes abbreviations. “A” means that the gene’s average threshold cycle is relatively high (> 30) in either the control or the test sample and is reasonably low in the other sample (< 30), “B” means that gene's average threshold cycle is relatively high (> 30), meaning that its relative expression level is low in both control and test samples, and the *p*-value for the fold-change is either unavailable or relatively high (*p* > 0.05), and “C” means that the gene’s average threshold cycle is either not determined or greater than the defined cut-off value, in both samples meaning that its expression was undetected, making this fold-change result erroneous and un-interpretable. SHRSP indicates spontaneously hypertensive stroke-prone rats, “HTX” heart transplantation

SHRSP vs Control				Control+HTX vs Control				SHRSP+HTX vs SHRSP				SHRSP+HTX vs Control+HTX			
Symbol	*P*-value	Regulation	Comments	Symbol	*P*-value	Regulation	Comments	Symbol	*P*-value	Regulation	Comments	Symbol	*P*-value	Regulation	Comments
Aifm1	0.001	1.35		Aifm1	0.714	-1.03		Aifm1	0.101	-1.08		Aifm1	0.014	1.29	
Akt1	0.000	-2.49		Akt1	0.507	-1.11		Akt1	0.212	-1.11		Akt1	0.000	-2.48	
Aox1	0.001	-2.93		Aox1	0.088	-1,69		Aox1	0.001	-1.62		Aox1	0.004	-2.80	
Apaf1	0.000	-2.12		Apaf1	0.119	-1.25		Apaf1	0.290	-1.11		Apaf1	0.000	-1.88	
Bak1	0.000	-1.83		Bak1	0.797	-1.03		Bak1	0.875	1.02		Bak1	0.001	-1.75	
Bax	0.004	-1.90		Bax	0.180	-1.26		Bax	0.121	-1.08		Bax	0.001	-1.63	
Bcl2	0.000	-1.70		Bcl2	0.073	-1.24		Bcl2	0.991	1.00		Bcl2	0.021	-1.37	
Bcl2l1	0.003	-2.05		Bcl2l1	0.090	1.53		Bcl2l1	0.710	-1.03		Bcl2l1	0.000	-3.22	
Bcl2l11	0.779	-1.05	A	Bcl2l11	0.265	1.25	B	Bcl2l11	0.605	1.06		Bcl2l11	0.127	-1.24	A
Bid	0.942	1.02		Bid	0.600	1.20		Bid	0.166	-1.11		Bid	0.240	-1.31	
Birc3	0.000	-2.23		Birc3	0.000	3.55		Birc3	0.000	2.95		Birc3	0.000	-2.68	
Birc5	0.830	-1.09	B	Birc5	0.724	-1.14	B	Birc5	0.463	-1.27	B	Birc5	0.517	-1.21	B
C4a	0.096	-1.85		C4a	0.061	-2,26		C4a	0.321	-1.29		C4a	0.882	-1.05	
Casp1	0.000	-2.43		Casp1	0.010	-1.70		Casp1	0.000	-1.61		Casp1	0.000	-2.30	
Casp12	0.000	-1.98		Casp12	0.005	-1.86		Casp12	0.021	-1.29		Casp12	0.059	-1.37	
Casp2	0.000	-3.18		Casp2	0.017	-1.82		Casp2	0.002	-1.65		Casp2	0.000	-2.88	
Casp3	0.006	-1.70		Casp3	0.264	1.18		Casp3	0.230	1.26		Casp3	0.029	-1.58	
Casp4	0.000	-2.18		Casp4	0.022	-1.83		Casp4	0.002	-1.39		Casp4	0.057	-1.65	
Casp6	0.000	-2.00		Casp6	0.010	-1.53		Casp6	0.030	-1.26		Casp6	0.004	-1.65	
Casp7	0.069	-1.30		Casp7	0.512	-1.09		Casp7	0.049	-1.23		Casp7	0.001	-1.48	
Casp8	0.001	-2.64		Casp8	0.222	-1.28		Casp8	0.916	-1.02		Casp8	0.001	-2.10	
Casp9	0.007	-1.37		Casp9	0.030	-1.46		Casp9	0.018	-1.30		Casp9	0.224	-1.22	
Cat	0.000	-2.24		Cat	0.256	-1,25		Cat	0.002	-1.25		Cat	0.002	-2.25	
Ccl11	0.079	3.47	A	Ccl11	0.225	-2.40	B	Ccl11	0.810	1.06		Ccl11	0.001	8.82	A
Ccl12	0.443	-1.32	A	Ccl12	0.047	-4.00	B	Ccl12	0.690	1.17		Ccl12	0.075	3.55	A
Ccl20	0.000	47.85	A	Ccl20	0.737	-1.10	C	Ccl20	0.453	1.10		Ccl20	0.000	57.74	A
Ccl24	0.000	-332.21	A	Ccl24	0.006	-1.79		Ccl24	0.458	-1.04	C	Ccl24	0.000	-193.76	A
Ccl3	0.000	-5.12		Ccl3	0.006	2.21		Ccl3	0.000	7.47		Ccl3	0.078	-1.51	
Ccl4	0.000	-5.84		Ccl4	0.052	1.67		Ccl4	0.000	5.21		Ccl4	0.021	-1.87	
Ccl5	0.000	-4.58		Ccl5	0.245	-1.24		Ccl5	0.642	1.10		Ccl5	0.000	-3.36	
Ccr2	0.000	-3.45		Ccr2	0.016	-2.14		Ccr2	0.005	-2.17		Ccr2	0.001	-3.49	
Ccr3	0.017	-4.87	B	Ccr3	0.114	-2.17	B	Ccr3	0.470	-1.54	B	Ccr3	0.026	-3.46	B
Ccs	0.409	-1.06		Ccs	0.037	-1,18		Ccs	0.028	-1.17		Ccs	0.458	-1.05	
Cd40lg	0.001	-6.10	B	Cd40lg	0.659	-1.31	B	Cd40lg	0.225	1.94	B	Cd40lg	0.222	-2.41	B
Ctsb	0.009	-1.40		Ctsb	0.427	-1,11		Ctsb	0.007	-1.20		Ctsb	0.004	-1.51	
Cxcr4	0.014	-1.63		Cxcr4	0.089	-1.41		Cxcr4	0.247	-1.29		Cxcr4	0.114	-1.49	
Cyba	0.001	-1.76		Cyba	0.038	-1,43		Cyba	0.016	-1.37		Cyba	0.003	-1.68	
Cycs	0.000	1.94		Cycs	0.843	1.03		Cycs	0.141	-1.10		Cycs	0.007	1.72	
Dhcr24	0.016	-1.32		Dhcr24	0.041	-1,33		Dhcr24	0.856	-1.02		Dhcr24	0.914	-1.02	
Duox1	0.147	-2.00	B	Duox1	0.664	1,26	B	Duox1	0.253	-1.63	B	Duox1	0.016	-4.12	B
Epx	0.007	-2.48	B	Epx	0.028	-2,32	B	Epx	0.013	-2.15	B	Epx	0.026	-2.29	B
Fadd	0.018	-1.44		Fadd	0.015	-1.50		Fadd	0.056	-1.24		Fadd	0.214	-1.18	
Fas	0.000	-2.50		Fas	0.085	-1.41		Fas	0.015	-1.32		Fas	0.000	-2.35	
Faslg	0.000	-10.44		Faslg	0.432	-1.22		Faslg	0.371	1.36		Faslg	0.000	-6.33	
Fos	0.365	1.49		Fos	0.000	31.62		Fos	0.000	36.09		Fos	0.052	1.70	
Gpx1	0.000	3.48		Gpx1	0.370	1,23		Gpx1	0.441	1.05		Gpx1	0.002	2.95	
Gpx4	0.001	1.28		Gpx4	0.425	1,07		Gpx4	0.856	-1.01		Gpx4	0.059	1.18	
Gpx7	0.033	-1.42		Gpx7	0.050	-1,54		Gpx7	0.093	-1.25		Gpx7	0.454	-1.15	
Gsr	0.148	-1.13		Gsr	0.105	-1,24		Gsr	0.004	-1.33		Gsr	0.109	-1.22	
Gstk1	0.001	-1.43		Gstk1	0.862	1,02		Gstk1	0.033	-1.20		Gstk1	0.000	-1.75	
Hmox1	0.718	1.08		Hmox1	0.000	5.30		Hmox1	0.004	2.46		Hmox1	0.022	-1.99	
Hspa4	0.521	1.02		Hspa4	0.017	1.33		Hspa4	0.288	1.06		Hspa4	0.055	-1.23	
Il10	0.701	-1.12	B	Il10	0.077	2.49	B	Il10	0.003	3.70	B	Il10	0.586	1.33	B
Il11	0.263	-1.66	B	Il11	0.042	2.14	B	Il11	0.036	3.79	B	Il11	0.894	1.07	B
Il13	0.000	-2.80	B	Il13	0.533	1.26	B	Il13	0.728	1.07	B	Il13	0.009	-3.30	B
Il15	0.000	-3.01		Il15	0.001	-2.77		Il15	0.000	-2.25		Il15	0.001	-2.45	
Il16	0.000	-3.01		Il16	0.014	-2.10		Il16	0.001	-2.02		Il16	0.001	-2.90	
Il3	0.005	-2.77	B	Il3	0.737	-1.10	C	Il3	0.332	-1.28	B	Il3	0.002	-3.22	C
Il4	0.000	-5.79	B	Il4	0.640	-1.16	B	Il4	0.378	1.16	B	Il4	0.000	-4.31	B
Il6	0.402	1.56	B	Il6	0.000	203.33	A	Il6	0.000	84.95	A	Il6	0.420	-1.54	
Il7	0.000	-5.52	A	Il7	0.009	-2.94	A	Il7	0.022	-3.63	B	Il7	0.003	-6.81	B
Il9	0.001	-2.92	B	Il9	0.737	-1.10	C	Il9	0.890	1.02	B	Il9	0.001	-2.61	B
Jun	0.003	-1.44		Jun	0.002	1.70		Jun	0.000	3.10		Jun	0.204	1.27	
Mapk1	0.001	-1.88		Mapk1	0.342	-1,13		Mapk1	0.034	-1.10		Mapk1	0.000	-1.83	
Mapk8	0.274	-1.10		Mapk8	0.702	1,05		Mapk8	0.670	-1.03		Mapk8	0.185	-1.18	
Mpo	0.000	-3.11	B	Mpo	0.999	1,00	B	Mpo	0.246	1.25	B	Mpo	0.009	-2.50	B
Ncf1	0.000	-2.33		Ncf1	0.938	-1,01		Ncf1	0.053	-1.28		Ncf1	0.000	-2.94	
Nfkb1	0.000	-1.40		Nfkb1	0.018	1,36		Nfkb1	0.160	1.11		Nfkb1	0.001	-1.73	
Nos2	0.001	-9.47	A	Nos2	0.003	2,27		Nos2	0.004	5.03	A	Nos2	0.000	-4.28	
Nox4	0.125	-1.46		Nox4	0.073	-1,96		Nox4	0.023	-1.57		Nox4	0.611	-1.17	
Noxo1	0.003	-3.92	B	Noxo1	0.549	-1,24	B	Noxo1	0.205	1.49	B	Noxo1	0.051	-2.12	B
Nqo1	0.106	-1.17		Nqo1	0.010	1,43		Nqo1	0.498	-1.03		Nqo1	0.000	-1.73	
Ptgs1	0.122	-1.27		Ptgs1	0.156	-1,36		Ptgs1	0.000	-1.59		Ptgs1	0.044	-1.48	
Sele	0.370	-1.30		Sele	0.020	2.46		Sele	0.007	2.64		Sele	0.576	-1.21	
Serpinb1b	0.454	1.25	B	Serpinb1b	0.808	1,07	B	Serpinb1b	0.001	-2.44	B	Serpinb1b	0.002	-2.09	B
Sod1	0.001	-1.26		Sod1	0.653	-1.03		Sod1	0.020	-1.08		Sod1	0.004	-1.31	
Sod2	0.456	-1.04		Sod2	0.183	1.10		Sod2	0.609	-1.03		Sod2	0.025	-1.18	
Sod3	0.777	-1.04		Sod3	0.000	-2.01		Sod3	0.224	-1.14		Sod3	0.000	1.69	
Srxn1	0.014	-1.56		Srxn1	0.000	3.54		Srxn1	0.000	2.65		Srxn1	0.004	-2.08	
Tgfb1	0.003	-2.12		Tgfb1	0.730	-1.07		Tgfb1	0.629	1.08		Tgfb1	0.005	-1.85	
Tnf	0.000	-8.96	A	Tnf	0.880	-1.03		Tnf	0.001	4.55	A	Tnf	0.023	-1.91	
Tnfrsf10b	0.001	-3.03		Tnfrsf10b	0.071	1,68		Tnfrsf10b	0.045	1.59		Tnfrsf10b	0.000	-3.21	
Tnfrsf1a	0.003	-1.70		Tnfrsf1a	0.000	2,11		Tnfrsf1a	0.006	1.81		Tnfrsf1a	0.002	-1.99	
Tnfsf10	0.000	-3.27		Tnfsf10	0.000	-3.95		Tnfsf10	0.000	-3.17		Tnfsf10	0.001	-2.63	
Tollip	0.001	-1.49		Tollip	0.304	-1.06		Tollip	0.317	-1.11		Tollip	0.000	-1.56	
Tp53	0.000	-1.95		Tp53	0.342	-1,19		Tp53	0.096	-1.14		Tp53	0.006	-1.87	
Tpo	0.001	-3.02	B	Tpo	0.623	-1.15	B	Tpo	0.307	-1.20	B	Tpo	0.001	-3.14	B
Txnrd1	0.201	-1.12		Txnrd1	0.004	1.80		Txnrd1	0.003	1.28		Txnrd1	0.014	-1.57	
Txnrd2	0.002	-1.59		Txnrd2	0.300	-1.14		Txnrd2	0.013	-1.29		Txnrd2	0.000	-1.81	
Ucp2	0.020	-1.30		Ucp2	0.824	-1.03		Ucp2	0.037	-1.20		Ucp2	0.002	-1.52	
Vimp	0.867	-1.01		Vimp	0.307	-1,08		Vimp	0.238	1.05		Vimp	0.100	1.12	
Xiap	0.003	-1.39		Xiap	0.478	-1,08		Xiap	0.006	-1.14		Xiap	0.006	-1.46	

#### After transplantation versus before transplantation

To determine the effects of heart transplantation-induced cardiac changes, 92 genes were surveyed. In normotensive hearts (control+transplanted versus control), among the tested genes, 18 genes were significantly altered compared to controls (10 genes were upregulated: *Birc3, Ccl3, Fos, Hmox1, Il11, Il6, Nos2, Sele, Srxn1, Tnfrsf1a* and 8 genes downregulated: *Cc12, Ccr2, Epx, Il15, Il16, Il7, Sod3, Tnfsf10*, Table 3, second column). In LVH hearts (SHRSP+transplanted versus SHRSP), among the tested genes, 20 genes were significantly altered (13 genes were upregulated: *Birc3, Ccl3, Ccl4, Fos, Hmox1, Il10, Il11, Il6, Jun, Nos2, Sele, Srxn1, Tnf* and 7 genes downregulated: *Ccr2, Epx, Il15, Il16, Il7, Serpinb1b, Tnfsf10*, Table 3, third column). However, there are 15 common genes (Table 3, second and third columns); therefore, in SHRSP rats, transplantation significantly altered 5 additional genes (J*un, Ccl4, Il10, Tnf, Serpnb1,* Table 3, third column). *Ccl4* and *Tnf*, which were down regulated in SHRSP rats compared to normotensive rats (Table 3, first column), were significantly upregulated after transplantation (Table 3, third column). The three altered genes *Tnfrsf1a, Sod3,* and *Ccl12* observed in control+transplanted versus control hearts were not found in SHRSP+transplanted versus SHRSP groups.

#### Graft with LVH versus control graft after transplantation

**Fig. 5:**
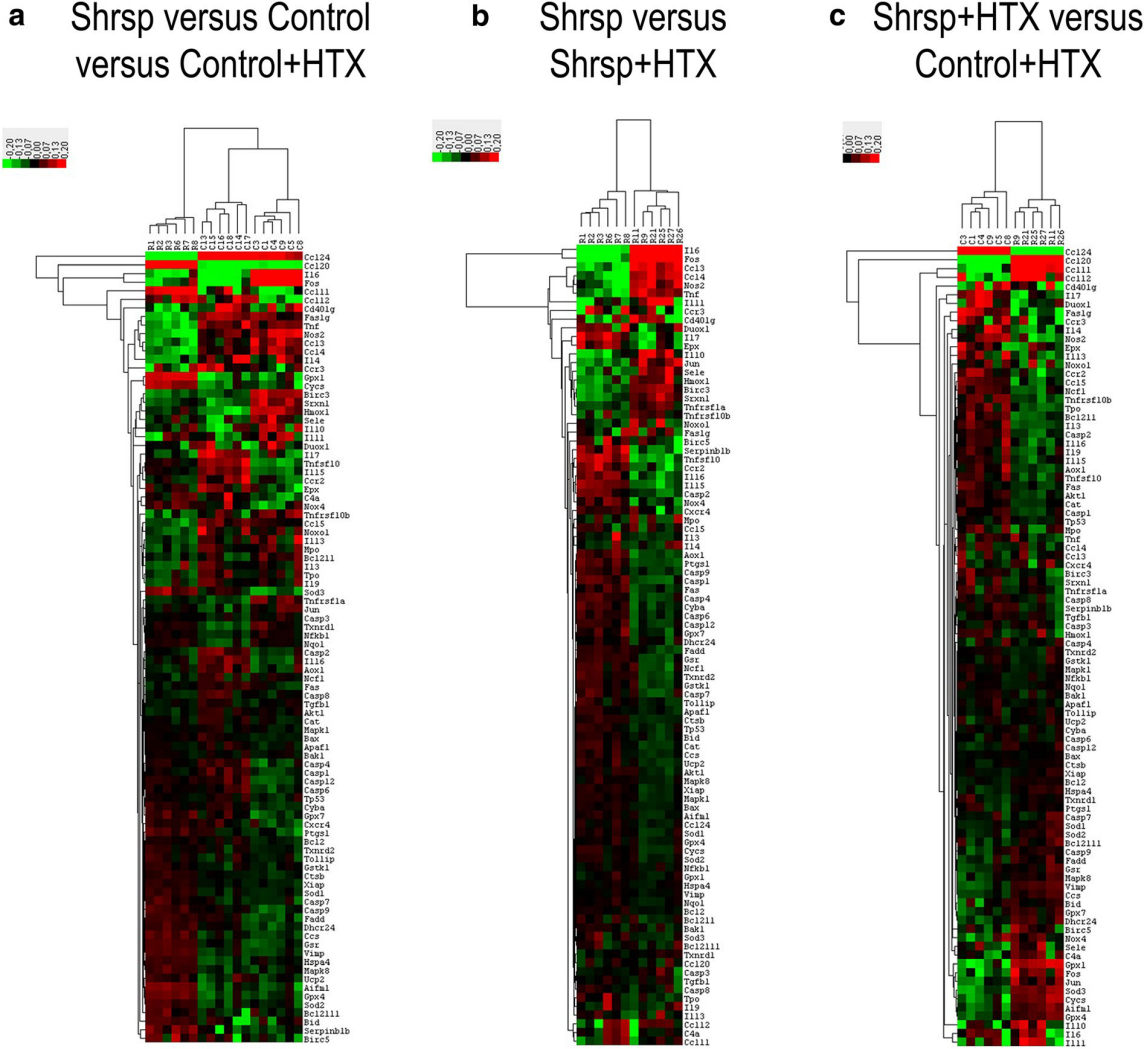
Changes in cardiac gene expression caused by left-ventricular hypertrophy in donors and their implication in heart transplantation. The expression of 92 genes involved in inflammation, apoptosis, and oxidative stress has been profiled in donors and in the graft after transplantation. Clustergrams create a heat map with dendograms to indicate which genes are co-regulated. Degrees of red and green indicate relatively high and low expression of the corresponding gene, respectively, and black squares denote genes equally expressed. (A) SHRSP versus Control and Control+HTX versus Control groups, (B) SHRSP+HTX versus SHRSP groups, and (C) Shrps+HTX veruss Control+HTX groups. The x-axis indicates the rat’s number [“C” corresponds to Control group, including six rats (C13, C14, C15, C16, C17, and C18), Control+HTX group, including six rats (C1, C3, C4, C5, C8, and C9), and “R” to SHRSP group, including six rats (R1, R2, R3, R6, R7, and R8), SHRSP+HTX group, including six rats (R9, R11, R21, R25, R26, and R27)], and the y-axis indicates the genes. SHRSP indicates spontaneously hypertensive stroke-prone rats, “HTX” heart transplantation. *n*= 6 rats/group

To determine the effects of both LVH and heart transplantation-induced cardiac changes, 92 genes were surveyed in the graft. The clustergrams create a heat map with dendrograms to indicate which genes are co-regulated (Fig. 5). Among the tested genes, 34 genes were significantly altered in SHRSP+transplanted rats compared to control+transplanted group (3 genes were upregulated: *Ccl11, Ccl20, Gpx1* and 31 genes downregulated: *Akt1, Aox1, Bcl2l1, Birc3, Casp1, Casp2, Casp8, Cat, Ccl24, Ccl5, Ccr2, Ccr3, Duox1, Epx, Fas, Faslg, Il13, Il15, Il16, Il3, Il4, Il7, Il9, Mpo, Ncf1, Nos2, Serpinb1b, Srxn1, Tnfrsf10b, Tnfsf10, Tpo*, Table 3 fourth column). However, among these 34 genes, 30 genes were common with those of Table 3, first column, i.e., the alteration is due to LVH and not to the transplantation. In other word, the additive effect of LVH and transplantation caused the alteration of 4 genes (*Ccl11, Duox1*, *Serpinb1b,* and *Srxn1,* Table 3, fourth column). Furthermore, the altered expression of 8 genes due to LVH was normalized after transplantation in SHRSP rats, i.e., they are likely not involved in graft changes after transplantation.

## Discussion

In this study, we investigated the effect of LVH on early post-transplant changes at functional levels and analyzed gene expression profiles in the hearts of 18-month-old SHRSP rats. To the best of our knowledge, this is the first report demonstrating that the alterations in donors, as consequence of hypertension and LVH, were not associated with LV graft dysfunction in the early phase of reperfusion after transplantation. Changes in the expression of antioxidant enzyme genes (*dual oxidase-1, serine peptidase inhibitor b1b*, and *sulfiredoxin-1*), the inflammatory cytokine *ccl11*, and reduced neutrophil infiltration were observed in the LVH hearts after transplantation. Taken together, decreased acute inflammatory response rather than reduced nitro-oxidative stress may, in part, explain the tolerance of donor hearts with LVH to ischemia/reperfusion injury during heart transplantation.

Efforts have been made to extend the acceptance criteria for hearts used for transplantation to allow the use of organs from “marginal” donors. However, there is little data regarding the transplantation of donor hearts with LVH [1, 8, 19] and the pathomechanisms of potential heart grafts from donors with LVH in cardiac transplantation have not been completely elucidated. Therefore, we characterized the effects of LVH in potential donor rats. In a previous study, Cingolani et al have shown that at 10 to 11 months of age, SHR have increased systolic performance accompanied by delayed relaxation and increased diastolic stiffness, using a pressure-volume conductance catheter system [2]. Our in vivo results confirmed enhanced cardiac mass leading to increased LV contractility (due to the adequate hypertrophic response) in the presence of diastolic dysfunction (due to an increase in wall thickness/fibrosis and alterations in relaxation/myocardial stiffness) in 18-month-old SHRSP donor rats compared to age-matched controls. Furthermore, the release of intracellular cardiac enzymes or markers in the circulation, such as troponin-T, lactate dehydrogenase, creatine kinase reflects cellular membrane damage and/or death of cardiomyocytes [18, 26]. Severe hypertension can cause relative ischemia due to the higher energy demand of the hypertrophied myocardium that is not met by the coronary circulation even in the absence of occlusion. In our study, hypertrophied hearts released more plasma levels of cardiac troponin-T and creatine kinase MB than control hearts, indicating cardiomyocyte injury. Furthermore, LVH increased the immunoreactivity for nitrotyrosine, a nitrooxidative stress marker. Finally, we profiled the myocardial expression of 92 genes in these rats. To the best of our knowledge, this is the first report describing that LVH altered the expression of 38 genes, involved in apoptosis, oxidative stress, and inflammatory response, in donor hearts. This may indicate a relationship between these genes and the cause of LVH in 18-month-old SHRSP rats.

Myocardial ischemia/reperfusion injury during heart transplantation impairs early graft function, contributing to adverse short-term [17] and long-term [32] graft outcome in the recipients. For the success of heart transplantation, a fast functional recovery of the transplanted donor heart is essential and an important determinant of the long-term outcome [27]. As profound hemodynamic changes occur during the early phase after transplantation, we focused our investigations on the early phase of reperfusion. Our data demonstrated that alterations observed in donors with LVH were not associated with LV graft dysfunction after heart transplantation. Involvement of inflammatory mechanisms in fibrotic processes is one of the main components of ventricular remodeling process [22]. Additionally, it is well recognized that cardiomyocyte specific apoptosis contributes to the transition from LVH to LV dysfunction [3]. Furthermore, oxidative stress has been identified as one of the key contributing factors in the development of cardiac hypertrophy [20]. Taken together, we used mRNA expression profiling to identify myocardial gene expression changes related to inflammation, apoptosis, and oxidative stress in donors and after heart transplantation. We profiled the expression of 92 genes. We showed that alterations of 38 genes were related to LVH (SHRSP versus normotensive rats), because their gene expression changes were independent from the effect of transplantation. The gene expression of 18 genes was, however, affected only by transplantation, independent of LVH (normotensive+transplanted versus normotensive rats). Furthermore, in SHRSP rats, transplantation significantly altered 5 additional genes (*jun, ccl4, Il10, tnf, serine peptidase inhibitor b1b*) (SHRSP+transplanted versus SHRSP). The additive effect of both LVH and transplantation was enough to alter the expression of *serine peptidase inhibitor b1b, dual oxidase-1, sulfiredoxin-1*, and *ccl11* (SHRSP+transplanted versus normotensive+transplanted). Additionally, the expression of *sulfiredoxin-1* was significantly up-regulated in the normotensive+transplanted group compared to normotensive group (+3.54) and in the shrps+transplanted group compared to SHRSP group (+2.65). LVH showed no effect on its gene expression. Our results suggest that the alteration of *sulfiredoxin-1* gene expression was the effect of transplantation alone. We did see a significant downregulation of *sulfiredoxin-1* gene expression (-2.08) in the SHRSP+transplanted group compared to normotensive+transplanted group. Although, at first glance, it seems that *sulfiredoxin-1* expression was only affected following transplantation, and LVH had no effect on its expression, it appears that LVH had an invisible effect on the tested genes. This became evident after an additional second injury. Sulfiredoxin-1, an endogenous antioxidant, has been shown to protect against simulated ischemia/reperfusion injury in cardiomyocytes [33] that is associated with reactive oxygen species-mediated cell death. Additionally, our PCR array results showed that *ccl11* expression was significantly increased (+8.8) in the shrps+transplanted group compared to normotensive+transplanted group. Zweifel et al. have shown that posttransplantation myocardial fibrosis correlated with eotaxin/CCL11 levels in rat models of transplantation [34]. They suggested that targeting eotaxin/CCL11 with monoclonal antibodies could reduce cardiac mast cell infiltration, possibly resulting in decreased myocardial fibrosis and improved contractile function after heart transplantation [34]. Neutrophils, circulating leucocytes, are usually the earliest cells to infiltrate transplanted tissue and their recruitment/activation plays an important role in transplant injury [28]. Our results show that whereas LVH has no effect on neutrophil infiltration in donor hearts, it decreases neutrophil count after transplantation. Surprisingly, we found that nitro-oxidative damage, evidenced by nitrotyrosine immunoreactivity in the transplanted hearts with LVH, was significantly increased.

From the clinical point of view, the aim of this study was to further elucidate the feasibility of optimal usage of “marginal” grafts in heart transplantation to identify and open-up new “druggable” therapeutic targets to increase the pool of donor organs.

In conclusion, alterations in donor hearts, as a consequence of hypertension and LVH, were not associated with LV graft dysfunction in the early phase of reperfusion after transplantation. Alterations in the expression of antioxidant enzyme genes (*dual oxidase-1, serine peptidase inhibitor b1b*, and *sulfiredoxin-1*) and the inflammatory cytokine *ccl11*, and decreased acute inflammatory response may, in part, explain the tolerance of donor hearts with LVH to ischemia/reperfusion injury during heart transplantation. It remains unclear whether other essential pathways may also take part in this effect. Further studies are needed to evaluate whether LVH in donors regresses in the late post-transplant period.

## Study limitations

First, our data suggests, without providing proof-of-concept, that the alteration of genes, including *dual oxidase-1, serine peptidase inhibitor b1b*, *sulfiredoxin-1* and *ccl11* may be possible targeting mechanisms when donor hearts with LVH are used. Second, in a clinical scenario, the use of hearts with LVH will be determined after careful selection, whereas in the present study LVH was left untreated. Finally, the possible adverse effects of immune cell activation, that can be triggered by heart transplantation, were not examined.

## Supplementary Information

ESM 1 (DOCX 15 kb)
