# Supplementary material for: Left-ventricular hypertrophy in 18-month-old donor rat hearts was not associated with graft dysfunction in the early phase of reperfusion after cardiac transplantation–gene expression profiling
Source: GeroScience. 2021 Apr 19;43(4):1995–2013. doi: 10.1007/s11357-021-00348-8 (PMC8492839; doi:10.1007/s11357-021-00348-8)
Supplement: Supplementary file 1 — (DOCX 15 kb) [file 11357_2021_348_MOESM1_ESM.docx]

**Online Table 1. List of genes and the pathways in which a given gene is involved.**

| **Gene Symbol** | **Official Full Name** |
| --- | --- |
|  | **Apoptosis- and cell survival-related genes** |
| Aifm1 | apoptosis-inducing factor, mitochondrion-associated 1 |
| Akt1 | v-akt murine thymoma viral oncogene homolog 1 |
| Apaf1 | apoptotic peptidase activating factor 1 |
| Bak1 | BCL2-antagonist/killer 1 |
| Bax | BCL2 associated X, apoptosis regulator |
| Bcl2 | BCL2, apoptosis regulator |
| Bcl2l1 | Bcl2-like 1 |
| Bcl2l11 | BCL2-like 11 (apoptosis facilitator) |
| Bid | BH3 interacting domain death agonist |
| Birc3 | baculoviral IAP repeat-containing 3 |
| Birc5 | baculoviral IAP repeat-containing 5 |
| C4a | complement component 4A (Rodgers blood group) |
| Casp1 | caspase 1 |
| Casp12 | caspase 12 |
| Casp2 | caspase 2 |
| Casp3 | caspase 3 |
| Casp4 | caspase 4 |
| Casp6 | caspase 6 |
| Casp7 | caspase 7 |
| Casp8 | caspase 8 |
| Casp9 | caspase 9, apoptosis-related cysteine peptidase |
| Cd40lg | CD40 ligand |
| Cycs | cytochrome c, somatic |
| Fadd | Fas (TNFRSF6)-associated via death domain |
| Fas | Fas cell surface death receptor |
| Faslg | Fas ligand (TNF superfamily, member 6) |
| Fos | FBJ osteosarcoma oncogene |
| Jun | jun proto-oncogene |
| Mapk1 | mitogen activated protein kinase 1 |
| Mapk8 | mitogen-activated protein kinase 8 |
| Nfkb1 | nuclear factor of kappa light polypeptide gene enhancer in B-cells 1 |
| Tnfrsf10b | tumor necrosis factor receptor superfamily, member 10b |
| Tnfrsf1a | tumor necrosis factor receptor superfamily, member 1a |
| Tp53 | tumor protein p53 |
| Xiap | X-linked inhibitor of apoptosis |
|  | **Genes involved in oxidative stress and antioxidant defense** |
| Aox1 | aldehyde oxidase 1 |
| Cat | catalase |
| Ccs | copper chaperone for superoxide dismutase |
| Ctsb | cathepsin B |
| Cyba | cytochrome b-245, alpha polypeptide |
| Dhcr24 | 24-dehydrocholesterol reductase |
| Duox1 | dual oxidase 1 |
| Epx | eosinophil peroxidase |
| Gpx1 | glutathione peroxidase 1 |
| Gpx4 | glutathione peroxidase 4 |
| Gpx7 | glutathione peroxidase 7 |
| Gsr | glutathione reductase |
| Gstk1 | glutathione S-transferase kappa 1 |
| Mpo | myeloperoxidase |
| Ncf1 | neutrophil cytosolic factor 1 |
| Nos2 | nitric oxide synthase 2, inducible |
| Nox4 | NADPH oxidase 4 |
| Noxo1 | NADPH oxidase organizer 1 |
| Nqo1 | NAD(P)H dehydrogenase, quinone 1 |
| Ptgs1 | prostaglandin-endoperoxide synthase 1 |
| Serpinb1b | serine (or cysteine) peptidase inhibitor, clade B, member 1b |
| Sod1 | superoxide dismutase 1, soluble |
| Sod2 | superoxide dismutase 2, mitochondrial |
| Sod3 | superoxide dismutase 3, extracellular |
| Srxn1 | sulfiredoxin 1 |
| Tpo | thyroid peroxidase |
| Txnrd1 | thioredoxin reductase 1 |
| Txnrd2 | thioredoxin reductase 2 |
| Ucp2 | uncoupling protein 2 (mitochondrial, proton carrier) |
| Vimp | VCP-interacting membrane protein |
|  | **Genes involved in inflammatory and anti-inflammatory response** |
| Ccl11 | chemokine (C-C motif) ligand 11 |
| Ccl12 | chemokine (C-C motif) ligand 12 |
| Ccl20 | chemokine (C-C motif) ligand 20 |
| Ccl24 | chemokine (C-C motif) ligand 24 |
| Ccl3 | chemokine (C-C motif) ligand 3 |
| Ccl4 | chemokine (C-C motif) ligand 4 |
| Ccl5 | C-C motif chemokine ligand 5 |
| Ccr2 | C-C motif chemokine receptor 2 |
| Ccr3 | chemokine (C-C motif) receptor 3 |
| Cxcr4 | chemokine (C-X-C motif) receptor 4 |
| Hmox1 | heme oxygenase (decycling) 1 |
| Hspa4 | heat shock protein 4 |
| Il10 | interleukin 10 |
| Il11 | interleukin 11 |
| Il13 | interleukin 13 |
| Il15 | interleukin 15 |
| Il16 | interleukin 16 |
| Il3 | interleukin 3 |
| Il4 | interleukin 4 |
| Il6 | interleukin 6 |
| Il7 | interleukin 7 |
| Il9 | interleukin 9 |
| Sele | selectin E |
| Tgfb1 | transforming growth factor, beta 1 |
| Tnf | tumor necrosis factor |
| Tnfsf10 | tumor necrosis factor superfamily member 10 |
| Tollip | toll interacting protein |
|  | **Housekeeping genes** |
| B2m | beta-2 microglobulin |
| Hprt1 | hypoxanthine phosphoribosyltransferase 1 |
